# Small molecule screen for inhibitors of expression from canonical CREB response element-containing promoters

**DOI:** 10.18632/oncotarget.7085

**Published:** 2016-01-30

**Authors:** Bryan Mitton, Katie Hsu, Ritika Dutta, Bruce C. Tiu, Nick Cox, Kevin G. McLure, Hee-Don Chae, Mark Smith, Elizabeth A. Eklund, David E. Solow-Cordero, Kathleen M. Sakamoto

**Affiliations:** ^1^ Department of Pediatrics, Stanford University, Stanford, CA, USA; ^2^ Medicinal Chemistry Knowledge Center, Stanford ChEM-H, Stanford, CA, USA; ^3^ Division of Hematology/Oncology, Department of Medicine, Northwestern University Feinberg School of Medicine, Chicago, IL, USA; ^4^ High-Throughput Bioscience Center, Department of Chemical and Systems Biology, Stanford University School of Medicine, Stanford, CA, USA

**Keywords:** small molecule screen, novel therapeutics, CREB

## Abstract

The transcription factor CREB (cAMP Response Element Binding Protein) is an important determinant in the growth of Acute Myeloid Leukemia (AML) cells. CREB overexpression increases AML cell growth by driving the expression of key regulators of apoptosis and the cell cycle. Conversely, CREB knockdown inhibits proliferation and survival of AML cells but not normal hematopoietic cells. Thus, CREB represents a promising drug target for the treatment of AML, which carries a poor prognosis. In this study, we performed a high-throughput small molecule screen to identify compounds that disrupt CREB function in AML cells. We screened ∼114,000 candidate compounds from Stanford University's small molecule library, and identified 5 molecules that inhibit CREB function at micromolar concentrations, but are non-toxic to normal hematopoietic cells. This study suggests that targeting CREB function using small molecules could provide alternative approaches to treat AML.

## INTRODUCTION

Successful treatment of Acute Myelogenous Leukemia (AML) remains a major clinical challenge. The 5-year overall disease survival rate for AML has held steady at less than 50% for the past 20 years, despite many efforts to intensify and broaden treatment regimens. The standard chemotherapeutics used for AML treatment are themselves associated with significant risks of morbidity and mortality, and while hematopoietic stem cell transplant is a viable option for therapy, this approach poses an additional set of risks for patients. For these reasons, it is critical to identify novel therapeutic targets and develop more effective, less toxic drugs for the treatment of patients with AML.

Our group and others have identified CREB (cAMP Response-Element Binding Protein) as one such potential therapeutic target. In a previous study, elevated CREB expression was observed in the majority of AML patients, and this independently predicted a worse prognosis and increased risk of relapse [1, 2]. Subsequent *in vitro* studies revealed that CREB overexpression in AML cells augments their growth rate and confers resistance to apoptosis [2]. In contrast, CREB knockdown inhibited AML cell proliferation and induced apoptosis, but had no effect on normal hematopoietic stem cells in mouse transduction/transplantation assays [3]. In addition, the expression of this potential drug target is typically much greater in AML cells compared to normal hematopoietic cells, the ‘parental’ tissue of this cancer. This suggests greater reliance on this transcription factor for AML cell homeostasis [1, 2, 4]. Given these data, we hypothesize that inhibition of CREB function may represent a novel, effective and targeted approach to treat AML.

Previous reports have described successful disruption of the association between CREB and its critical transcriptional co-activator CBP [5]. Post-translational modification of CREB including phosphorylation, acetylation, and SUMOylation, are critical for its function [6]. CREB also differentially binds as a homodimer or heterodimer with members of the ATF transcription factor family, resulting in differential gene expression based on the cellular context [6]. These observations demonstrate that numerous CREB functionalities could be selected for targeted disruption by a small molecule. Fortunately, the advent of high-throughput screening has facilitated exploration of a wide array of chemical moieties in search of molecules which may disrupt any of these diverse processes, even in the absence of *a priori* knowledge of which of these processes are most important for cellular homeostasis.

Thus, in this study, we performed a small molecule screen in search of compounds capable of disrupting CREB-driven transcription in AML cells. To this end, we screened 114,124 candidate compounds from the compound library available at the Stanford University High-Throughput Bioscience Center. This collection was assembled from several commercial vendors, including ChemDiv, Specs, and Chembridge, and possesses drug-like qualities [7]. These compounds were first tested for their ability to specifically disrupt CREB-driven expression of a reporter gene in KG-1 cells. Selected compounds that passed this initial screen were validated and examined for their ability to selectively kill AML cells, but not normal hematopoietic cells, *in vitro*. Our study yielded five structurally distinct and novel molecules that inhibit the growth of AML cells at micromolar concentrations yet are less toxic to normal hematopoietic cells than doxorubicin, a conventional chemotherapeutic for AML. This study thus describes the means for developing an assay to screen for “transcription factor therapeutics”, and successfully identified unique chemical candidates with potential for drug development in the future.

## RESULTS

### High-throughput screen identifies candidate CREB-inhibiting compounds

Since CREB represents a promising therapeutic target for AML, a high throughput screen was conducted, followed by validation assays, to identify potentially novel disruptors of CREB function. To identify compounds that specifically disrupt CREB-driven transcription, we generated a KG-1 AML cell line expressing Firefly luciferase in a CREB-specific manner (KG-1 CRE), and a separate KG-1 cell line expressing Firefly luciferase under the control of the CMV promoter (KG-1 CMV), as described in *Methods*. The Stanford University's High-Throughput Bioscience Center's (HTBC) Small Molecule Library was first screened against KG-1 CRE cells for 24 hours. This incubation period empirically allowed sufficient time for reporter gene transcriptional downregulation, but preceded AML cell death. Of 114,124 compounds screened, 1481 reduced KG-1 CRE luciferase activity by greater than 45% (Table 1). For this phase of high-throughput screening, the Z'-factor was = 0.6, coefficient of variance (CV) = 15% with no significant edge or drift effects. Compounds that appeared as being active in greater than two thirds of previously run luciferase assays in the HTBC, with other transcription factor binding sites, were eliminated as likely luciferase inhibitors, while compounds that appeared as toxic compounds in greater than two thirds of previously run human cell viability assays were ruled as non-specific toxic and eliminated from further studies.

**Table 1:** Summary of high throughput screening hit results

HTS Luciferase Inhibition	Number of Hits	Luminescence Inhibitors	Toxic	Selected for Extended Analysis
> 70%	429	122	75	229
60–70%	234	26	63	145
50–60%	417	31	101	283
45–50%	401	8	70	323
Total	1481	187	309	980

KG-1 CRE cells were used to screen the Stanford Small Molecule Library of 114,124 compounds. Shown are the number of putative hits, shown by decile of luciferase inhibition, and the number selected for further study based on physicochemical properties.

**Figure 1:**
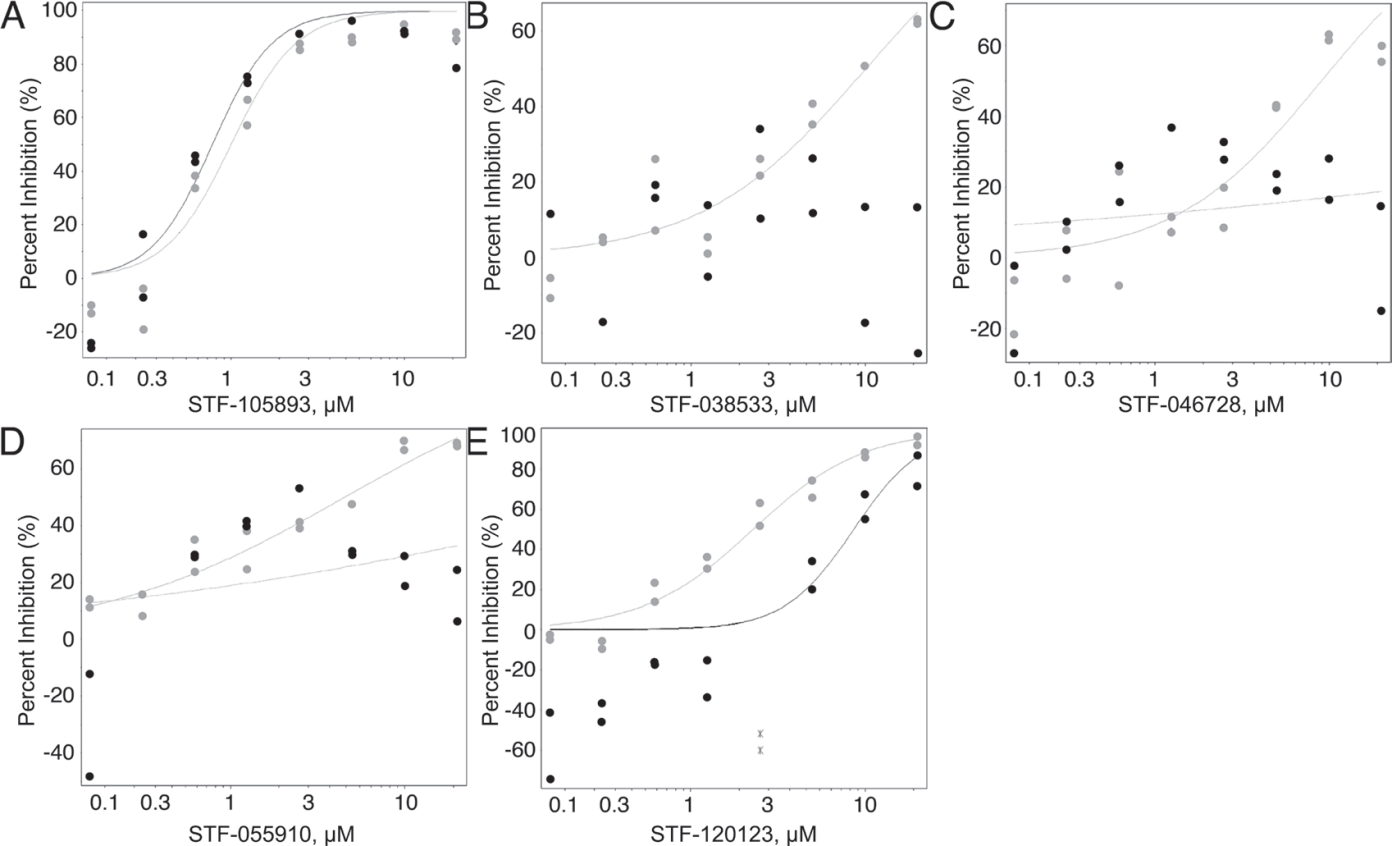
Candidate compound counter-selection From 114, 124 compounds, 980 were selected for counter-screening against KG-1 CMV cells. This was done in order to discriminate between compounds that only suppressed CREB-driven luciferase activity (grey) and those that non-specifically suppressed luciferase (black) in both AML cell lines. (**A**) Representative graph of compounds that reduced luciferase activity in both cell lines equally. (**B**–**E**) Representative graphs of compounds with an IC_50_ 2-fold higher in KG-1 CMV cells compared to KG-1 CRE cells (were more potent against KG-1 CRE cells) were selected for further study. From 980 compounds, 23 were selected for further study.

Following this, 8-point dose-response curves for each concentration in duplicate were generated for 980 selected compounds using both the KG-1 CRE and KG-1 CMV cell lines. For the purposes of screening, this approach facilitated the inclusion of compounds, which selectively inhibited CREB-driven luciferase expression for further study, and the exclusion of those compounds which were luminescence inhibitors or which were non-selectively toxic. This extended screening identified 59 compounds with IC_50_ value ratios of > 2 in KG-1 CRE vs. KG-1 CMV cells, suggesting CREB-specific transcriptional disruption (data not shown). With 3 exceptions, all of these compounds had IC_50_ values of < 10 μM in KG-1 CRE cells. Based on each compound's absolute IC_50_, the ratio of IC_50_ values in each KG-1 cell line, and each compound's physico-chemical properties and potential drug-like structure, 23 of these compounds were selected for further study (Figure 1 and Table 2).

**Table 2:** Summary of compound potency and selectivity in HTS screen

Compound	IC_50_ (μM)	IC_50_ Selectivity Ratio
STF–041861	1.2	3.7
STF–030418	1.3	3.1
STF–041268	1.6	2.9
STF–120123	2.3	3.8
STF–022005	2.3	5.6
STF–021727	3.1	3.9
STF–020294	3.3	3.1
STF–036803	4.0	5.1
STF–086043	4.4	4.6
STF–055910	4.5	4.4
STF–047300	4.6	4.3
STF–046536	4.7	4.3
STF–020571	4.8	5.2
STF–033399	5.4	3.3
STF–057955	5.9	5.1
STF–035696	6.8	2.9
STF–082587	7.4	3.3
STF–022897	8.1	3.1
STF–022933	8.2	2.4
STF–046728	9.0	2.2
STF–014140	9.0	2.2
STF–126794	9.7	2.1
STF–038533	10.0	2.0

KG-1 CRE and KG-1 CMV cells were treated with 8 concentrations with each of 980 compounds; the results for 23 compounds, which showed micromolar potency and an IC_50_ selectivity ratio > 2 in KG-1 CRE vs. KG-1 CMV AML cells are shown here. These compounds were selected for further validation and activity studies in AML cells.

### Validation of CREB inhibitors

**Figure 2:**
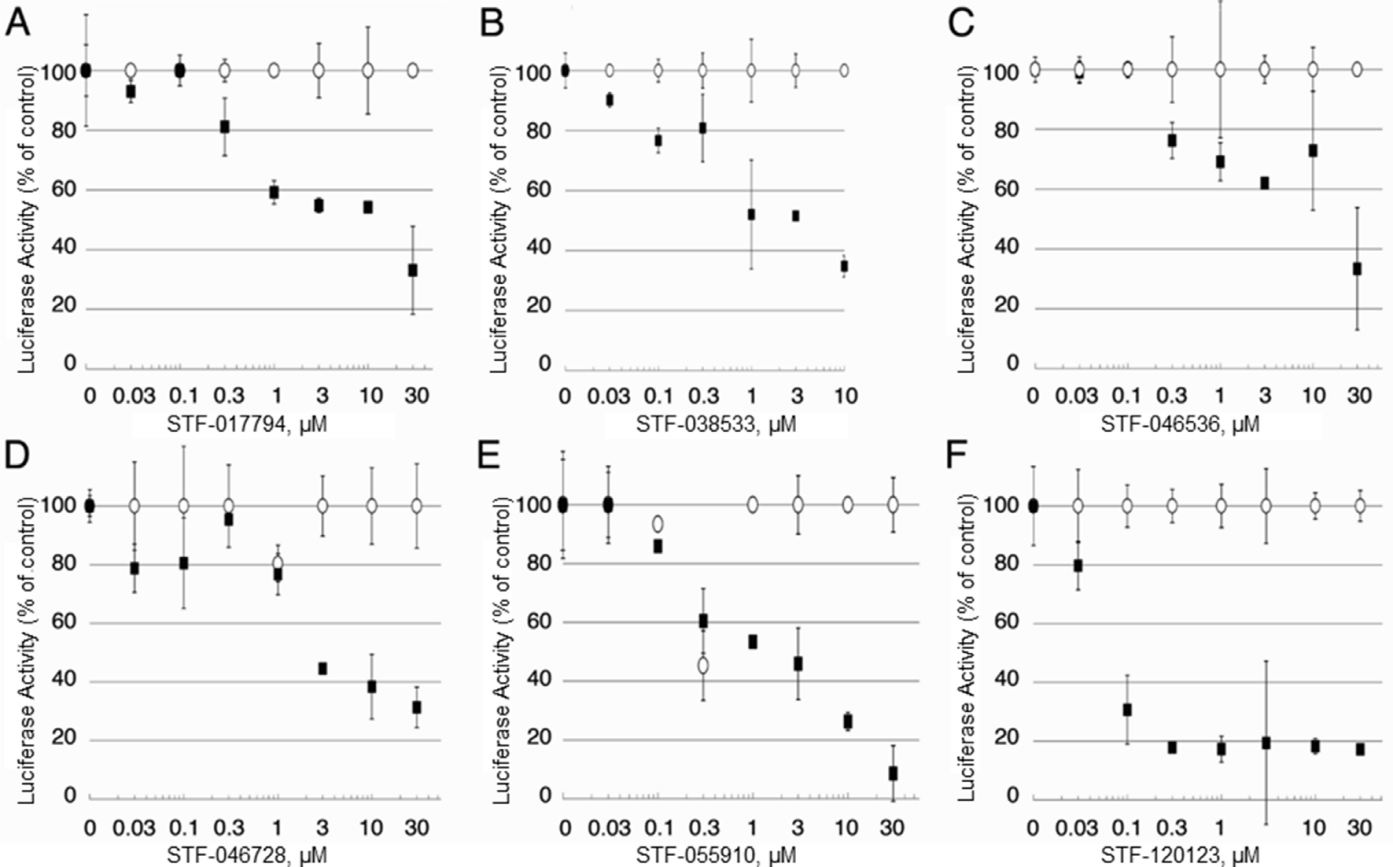
Candidate compound selectivity for CREB-driven vs. non-CREB-driven luciferase expression (**A**–**F**) The 23 compounds with putative selectivity for CREB-driven luciferase expression were tested in upscaled luciferase assays using KG-1 CRE and KG-1 CMV AML cells. In these extended assays, 6 of 23 compounds (STF-017794, STF-038533, STF-046536, STF-046728, STF-055910 and STF-120123) showed very little activity in suppressing non-CREB-driven luciferase expression (white), but were able to suppress CREB-driven luciferase expression (black) with at least three concentrations of compound.

To confirm the results of the high-throughput screen, the selectivity of the 23 candidate compounds was first validated in scaled-up luciferase activity experiments, performed in duplicate (Figure 2). KG-1 CRE and KG-1 CMV cells were treated with compound concentrations ranging from 30 nM to 30 μM for 24 hours. As shown, this validation assay was able to discriminate between selective compounds and inactive or insufficiently selective compounds. Of 23 candidates, six compounds demonstrated sufficient selectivity for further study, with specific suppression of luciferase activity in KG-1 CRE cells but not KG-1 CMV cells at a minimum of three concentrations.

**Figure 3:**
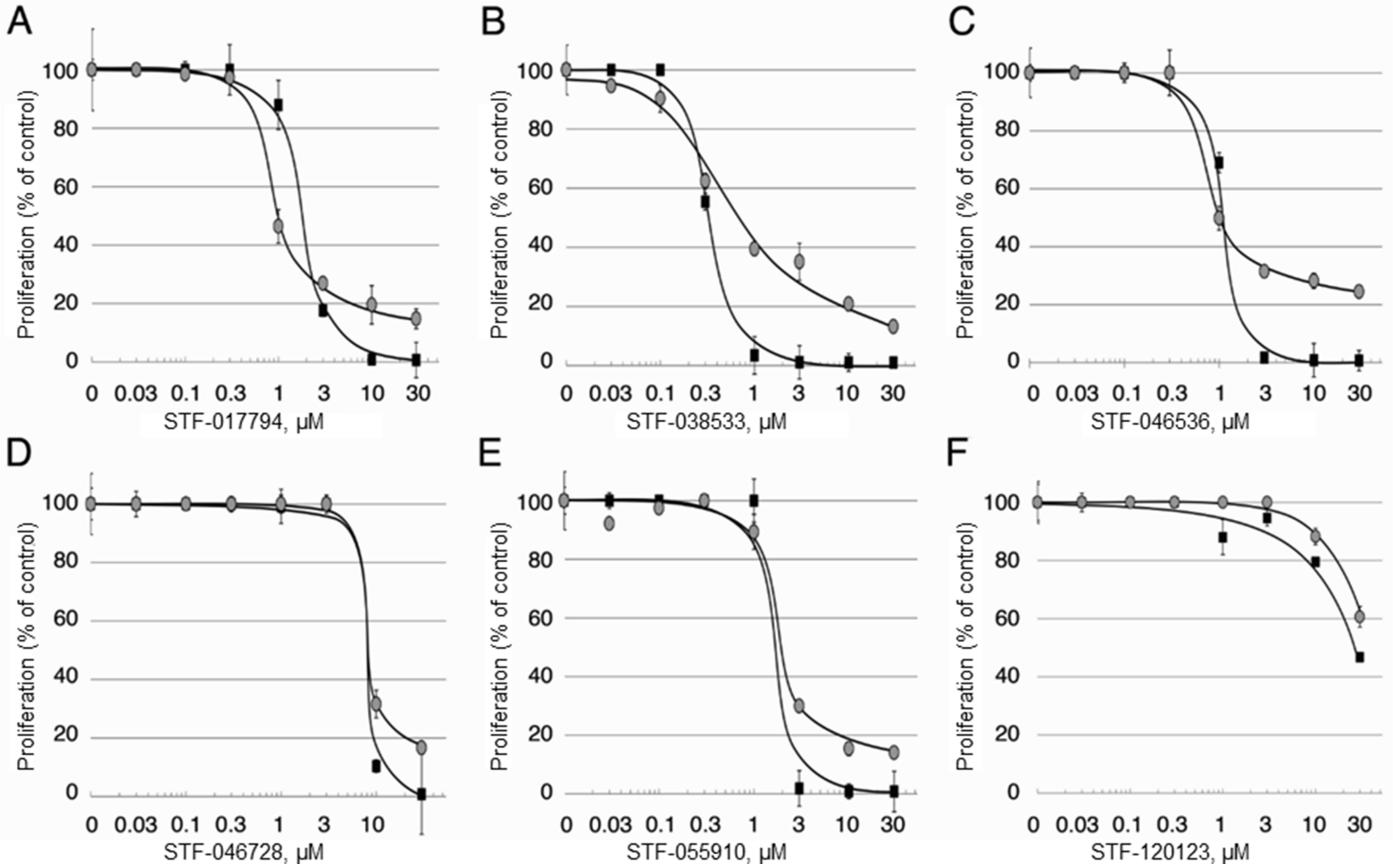
Efficacy of candidate compounds against AML cells *in vitro* The efficacy of the 6 candidate compounds inhibiting cell proliferation was tested against two well-characterized AML cell lines as described in *Materials and Methods*.

To examine whether these compounds were able to kill AML cells *in vitro*, well-characterized HL-60 and KG-1 cells were treated with compound concentrations ranging from 30 nM to 30 μM for 72 hours. Dose-response curves for each of these six compounds were generated using the Promega CellTiter Glo Viability Assay. Five compounds showed low micromolar IC_50_ values when tested against unmodified HL-60 and KG-1 AML cell lines for 72 hours (Figure 3 and Table 3). Compound STF-038533 showed an IC_50_ of 410 nM, a potency comparable to that of rationally-designed drugs in clinical use such as sorafenib or ABT-737 [10, 11]. In contrast, compound STF-120123, which showed excellent selectivity in decreasing CREB-driven luciferase activity in KG-1 CRE vs. KG-1 CMV cells, showed low potency in these assays. Nonetheless, the potency of the five best compounds compare favorably to potency values reported for conventional chemotherapeutics used in AML treatment regimens, including cytarabine, doxorubicin and etoposide, when tested similarly *in vitro*. The structures of each library hit are shown along with a summary of the cell-based IC_50_ values (Table 3). Various chemotypes are represented, reflecting the diversity of the library.

**Table 3:** Compound structures and data summary

Compounds		Cell Assay IC_50_ Values (μM)		
Number	Structure	KG-1 Cre-Luc^*a*^	KG-1 Viability^*b*^	HL-60 Viability^*c*^
STF–038533	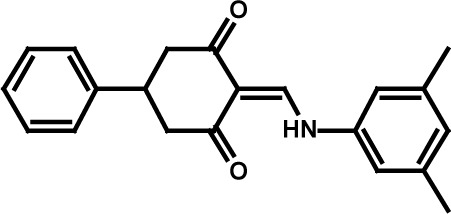	2.4	0.83	0.41
STF–046536	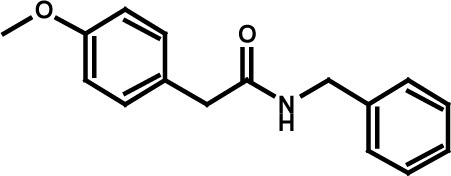	15.5	0.72	1.1
STF–017794	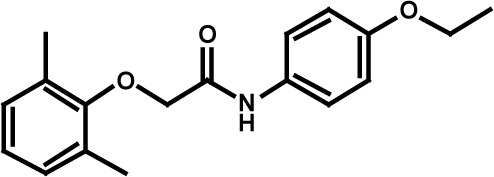	2.9	0.99	1.3
STF–055910	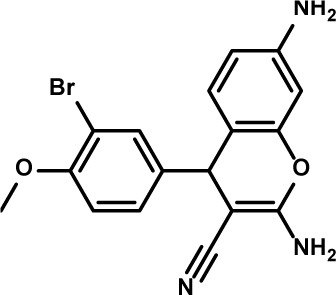	1.5	1.6	1.8
STF–046728	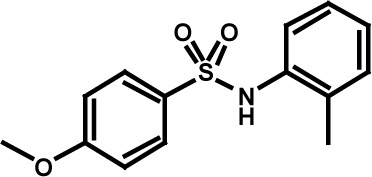	4.7	4.8	5.4
STF–120123	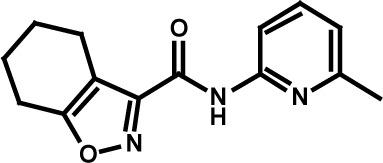	0.058	> 10	> 10

Structures of each compound along with IC_50_ values are shown. (a) IC_50_ values for each compound inhibiting Cre-Luc expression as determined from the experiment in Figure 2. (b) and (c) IC_50_ values for each compound inhibiting cell viability as determined from the experiment in Figure 3.

### Non-toxicity of CREB inhibitors

**Figure 4:**
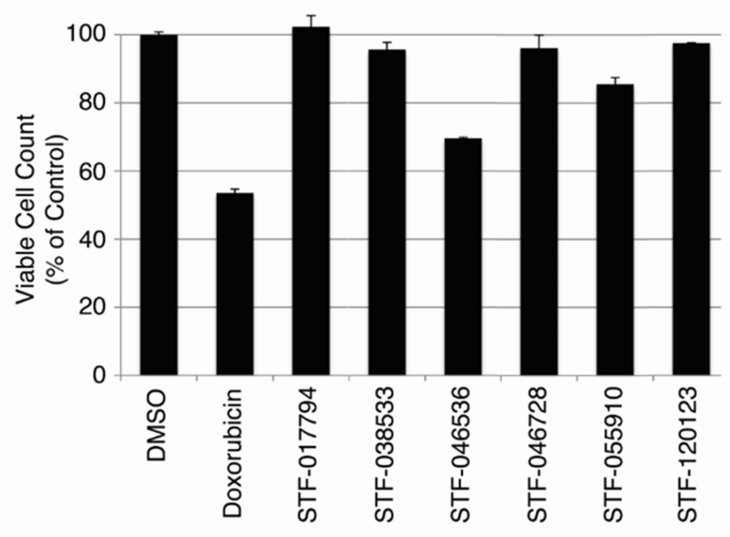
Toxicity of candidate compounds to normal bone marrow cells *in vitro* To evaluate the potential toxicity of these compounds against non-cancer cells, cultured bone marrow cells from human donors were treated with each of 6 candidate compounds (10 μM) for 72 hours. Doxorubicin (3.8 μM) was used as a positive control. The viable cell count, shown as a percent of viable cells remaining after DMSO (vehicle) treatment, remained > 95% of control for cells treated with STF-017794, STF-038533, STF-046728 and STF-120123.

**Figure 5:**
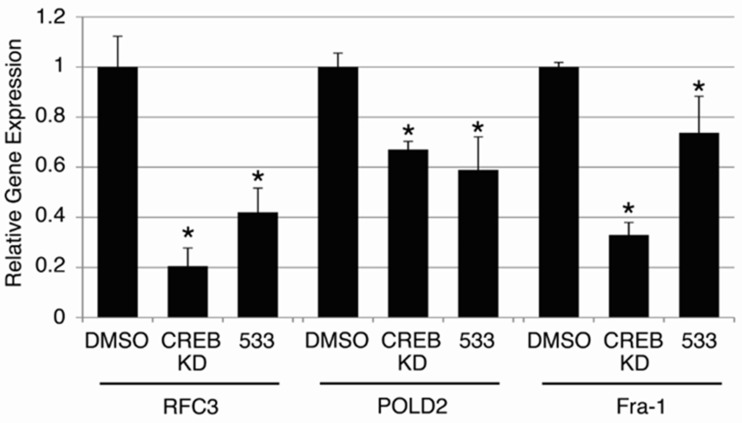
On-target effects of candidate compound STF-038533 To assess whether compound STF-038533(533) exerted ‘on-target’ effects on validated CREB target genes, the expression of *RFC3*, *Fra-1* and *POLD2* were examined following 24 hours of treatment with STF-038533 (10 μM) and compared to KG-1 cells in which CREB expression was reduced by shRNA(CREB KD). Each of these genes exhibited significantly reduced expression compared to control cells, treated with DMSO (*p < 0.05).

Since CREB knockdown decreased AML cell growth, but not that of normal hematopoietic cells in transplantation assays, we wished to test whether these putative CREB-inhibiting compounds were also non-toxic to normal hematopoietic cells. Normal hematopoietic cells were cultured in standard media as described in *Materials and Methods*, and treated with 10 μM of each of the six candidate compounds for 72 hours. Doxorubicin (2 mg/mL, 3.8 μM), a standard chemotherapeutic agent used for AML treatment, was used as a control. The viable cell count remaining after 72 hours in each treatment condition is expressed as a percentage of cells present following treatment with 0.1% DMSO solvent (Figure 4). Doxorubicin caused a 46.5 ± 0.77% reduction in viable cell count following 72 hours of treatment. Compounds STF-046536 and STF-055910 caused viable cell count reductions of 30.5 ± 1.16% and 14.5 ± 2.91%, respectively, while all other compounds reduced the viable cell count by < 5%. Thus, these compounds possess favorable therapeutic indices, with better *in vitro* potency and lower toxicity than doxorubicin. Given the high potency (low IC_50_ value), selectivity and non-toxicity of compound STF-038533, this molecule was analyzed for its ability to reduce CREB target gene transcription. The *RFC3*, *POLD2* and *Fra-1* genes each possess CRE elements within 200 bp of their transcription start sites, and previous work supports the importance of CREB in regulating their transcription [8, 12]. RT-PCR data show that the transcription of these genes in KG-1 cells treated with 10 μM STF-038533 for 24 hours was reduced compared to DMSO-treated cells. KG-1 cells with CREB knockdown using shRNAs were used as a positive control (Figure 5).

## DISCUSSION

Recent preclinical reports have demonstrated the efficacy of targeting transcription factors in specific cancers [13–16]. The association of CBP with β- and γ-catenin has been targeted using a small molecule, and this strategy was effective against both primary and relapsed ALL in mice [13]. Another group demonstrated the efficacy of targeting the mutant fusion transcription factor CBPβ-SMMHC, which drives inv(16)^+^ AML [15]. The association between menin and MLL fusion proteins, which drives subtypes of both AML and ALL, has also been successfully targeted using a small molecule [14]. These studies demonstrate the potential of transcription factor-directed therapy, and encourage further development of these novel candidate compounds for eventual clinical use. In this study, we employed a high-throughput screening strategy, which yielded five chemically unique compounds, which demonstrated promising *in vitro* potencies and efficacies and showed little to no toxicity to normal hematopoietic cells compared to doxorubicin.

In summary, the data presented here suggest that the development of small molecules that target CREB could lead to novel approaches to treat AML. Although the compounds identified require structure-activity relationship-based optimization, characterization of their specific mechanism(s) of action, and *in vivo* testing prior to their entry into clinical trials, our data encourage further studies of the advantages of targeting this transcription factor.

## METHODS

### AML cell lines

AML cell lines were purchased from ATCC and maintained with IMDM (Gibco) supplemented with 10% FBS (Fisher Scientific) and 1% PSG (Gibco). KG-1 is a well-characterized human AML cell line commonly used to study novel therapies for AML. KG-1 cells were established from a patient with AML and represents an early stage of hematopoietic differentiation. KG-1 cells are also p53 deficient. HL-60 is an AML cell line that was derived from a patient with acute promyelocytic leukemia in 1977. The cells can be induced to differentiate into granulocytes or monocyte/macrophages. HL-60 cells also overexpress myc. These two cell lines have been extensively used to test novel molecules to potentially treat AML.

**Figure 6:**
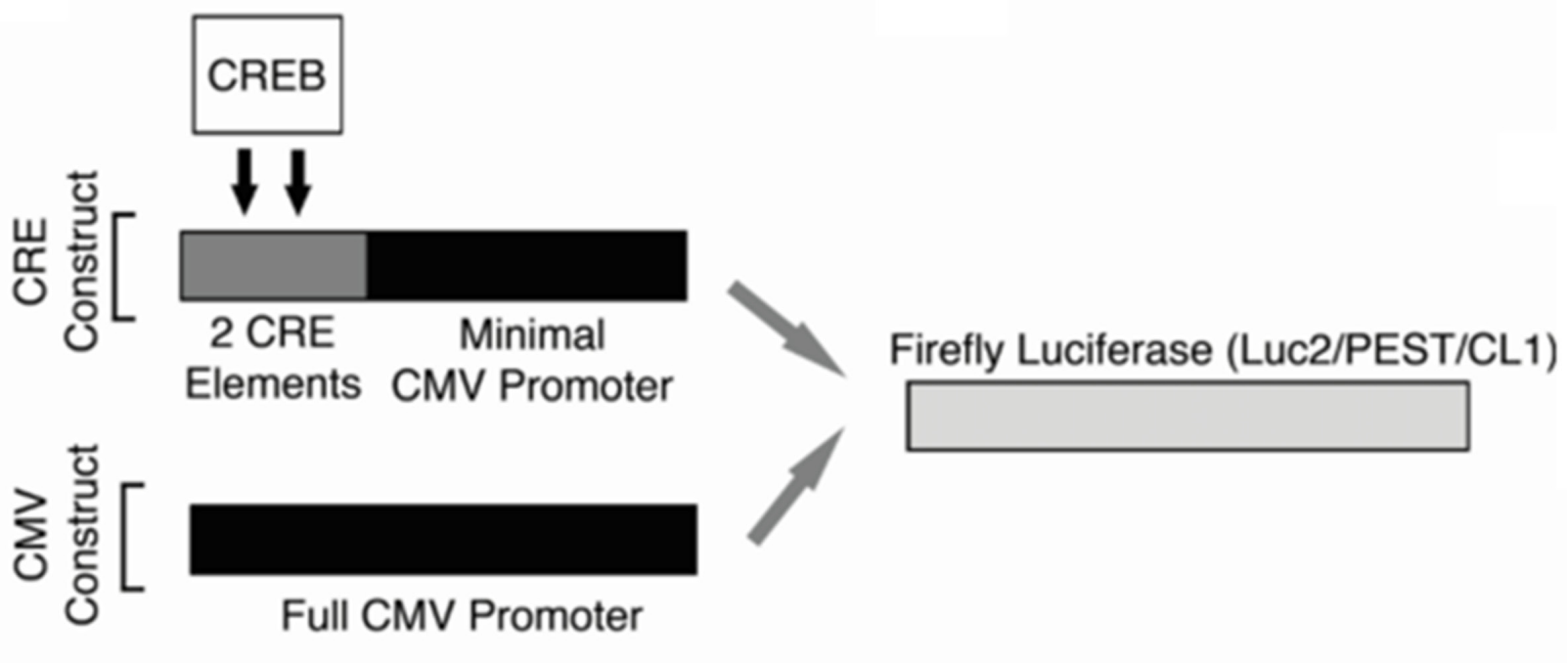
High-throughput screening strategy Promoter schematics for the two KG-1 cell lines generated for use in the high-throughput screen. For both cell lines, a Firefly luciferase gene containing two C-terminal protein destabilizing sequences (hCL1 and h PEST) was used in order to reduce this protein's half-life and facilitate early changes in the expression of this reporter gene. The promoter for CREB-specific expression of luciferase was composed of two ‘CRE’ elements, which are the canonical CREB DNA-binding sequences, placed within −200 of the ATG start site and 5′ to the CMV minimal promoter (KG-1 CRE cells). For non-specific luciferase expression, the full CMV promoter (KG-1 CMV cells) was placed 5′ to the luciferase gene.

Cell counts and viability were determined using the Vi-CELL XR Cell Viability Analyzer (Beckman Coulter). For the high-throughput screen, lentiviral gene delivery was used to create two KG-1 cell lines designed to express Firefly luciferase in either a CREB-specific or non-specific fashion (Figure 6). Cells were cultured in media containing 10% serum containing growth factors. Under these conditions, CREB is phosphorylated and activated. The promoter for CREB-specific expression of luciferase was composed of two ‘CRE’ elements, which are the canonical CREB DNA-binding sequences, placed within −200 of the ATG start site and 5′ to an attenuated CMV promoter (KG-1 CRE cells). Non-specific luciferase expression was driven by the standard full CMV promoter (KG-1 CMV cells). To create lentiviruses, each of these promoter cassettes were first assembled and inserted 5′ to the Firefly luciferase gene harbored in the pGL4.22 [luc2CP/Puro] vector (Promega). This Firefly luciferase gene sequence contains two C-terminal protein destabilizing sequences, hCL1 and hPEST, resulting in a short protein half-life. These sequences facilitated early measurement of luciferase transcriptional downregulation. Lentiviruses were generated as previously described [9].

### High-throughput screening

For this HTS to discover small molecules that specifically disrupt CREB function, we screened ∼114,000 compounds from the compound library at the Stanford University High-Throughput Bioscience Center. These compounds are drug-like and were assembled from various commercial sources including, ChemDiv (60 K), Specs (30 K), and Chembridge (23.5 K). The compound library is arrayed in 384-well plates at a concentration of 5 mM in 100% DMSO, except for the Chembridge library which is at a concentration of 2 mg/mL in 100% DMSO. The HTS was performed by plating 25,000 KG-1 CRE cells in 50 μL IMDM + 10% FBS + 1% PSG media per well in 384-well solid-white tissue cultured treated plates, and adding ∼100 nL of candidate compounds using a V & P Scientific Pin Tool attached to a Sciclone ALH3000 (CaliperLS, now Perkin Elmer) resulting in a final concentration of ∼10 μM or ∼4 ug/mL for the Chembridge library. Each compound was only screened once. The cells were incubated for 24 hours at 37°C/5% CO_2_, in an automated incubator (Liconic STX200) and subsequent luciferase activity was assessed by adding 10 μL of a 1:3 mixture of BrightGlo luciferin reagent (Promega) and media by an offline Multidrop reagent dispenser (Labsystems) and reading luciferase activity after 10 minutes incubation at room temperature on a Tecan Infinite M1000 PRO with integrated stackers, with 0.1 sec signal integration time. The 24-hour incubation time was selected because this allowed sufficient time for luciferase transcriptional downregulation, but preceded anticipated decreases in cell viability resulting from CREB inhibition. Compounds, which caused > 45% reduced luciferase activity in KG-1 CRE cells were selected for further screening (see results for more details). Hits were manually picked from stock supplies and diluted to 100 μM in media (no serum) and then serially diluted 1:1 in media (no serum) using the High-Volume Head on the Sciclone ALH3000 (CaliperLS, now Perkin Elmer). 10 μL of the serially diluted compounds was added to the 40 μL of the cells (25 K/well). An eight-point dose-response curve was generated for both KG-1 CRE and KG-1 CMV cell lines using these methods for selected compounds and performed in duplicate. The final compound concentrations used for this secondary screen were 20, 10, 5, 2.5, 1.25, 0.625, 0.3125, and 0.156 μM.

### Luciferase validation and cell viability assays

For candidate compound validation experiments, KG-1 CRE or KG-1 CMV cells were plated at a density of 2 × 10^5^ cells/mL in a 96-well plate and incubated with concentrations of candidate compounds ranging from 30 nM to 30 μM for 24 hours. Following this, 20 μL of BrightGlo luciferin was added to each well and the luciferase activity was measured using a Biotek Synergy H1 spectrophotometer, 10 second integration time.

For candidate compound activity assays in AML cells, unmodified KG-1 or HL-60 cells were plated in 96-well plates at a density of 2 × 10^5^ cells/mL and incubated with concentrations of candidate compounds ranging from 30 nM to 30 μM for 24 hours. The Promega CellTiter-Glo Luminescent Viability Assay (Promega) was used as per manufacturer's instructions to measure viable cell counts after 72 hours. This timepoint was selected, as this is the typical timepoint at which conventional chemotherapeutics demonstrate maximal efficacy in *in vitro* assays.

### RT-PCR

Following 24 hours of treatment with selected candidate compounds (10 μM), KG-1 cells were harvested and total RNA was isolated using the Aurum RNA Isolation Mini Kit (Bio-Rad). The iScript cDNA Synthesis Kit was used to prepare samples for qPCR (Bio-Rad). PCR was performed on the CFX384 Real-Time System (Bio-Rad) and results were analyzed using the Livak method [9].

### Human patient samples

Normal human patient bone marrow samples were purchased from StemCell Technologies. Bone marrow cells were cultured in DMEM plus 20% FBS and 1% PSG, supplemented with recombinant GM-CSF (20 ng/ml), G-CSF (20 ng/ml), SCF (50 ng/ml), IL-3 (20 ng/ml), and IL-6 (10 ng/ml). For candidate compound activity assays, cells were plated at 1 × 10^5^ cells/ml in a 12-well plate, supplemented with a range of concentrations of candidate compound, for 24 hours. Cell counts and viability were determined using the Vi-CELL XR Cell Viability Analyzer (Beckman Coulter).

### Statistical analysis

Primary screening and secondary dose response data were analyzed using Assay Explorer (MDL, now Biovia). Z' were calculated for all plates and plates that failed validation (Z' < 0.5) were re-screened. Unless noted, all experiments were performed in triplicate, and Student's *t*-test was used to assess experimental mean values for statistically significant differences, with *p*-values of < 0.05 deemed statistically significant.
